# Deregulation of the miRNAs Expression in Cervical Cancer: Human Papillomavirus Implications

**DOI:** 10.1155/2013/407052

**Published:** 2013-12-31

**Authors:** Yazmín Gómez-Gómez, Jorge Organista-Nava, Patricio Gariglio

**Affiliations:** ^1^Instituto de Fisiología Celular (IFC), Universidad Nacional Autónoma de México (UNAM), 04510 México, DF, Mexico; ^2^Departamento de Genética y Biología Molecular, Centro de Investigación y de Estudios, Avanzados, 07360 México, DF, Mexico

## Abstract

MicroRNAs (miRNAs) are a class of small non coding RNAs of 18–25 nucleotides in length. The temporal or short-lived expression of the miRNAs modulates gene expression post transcriptionally. Studies have revealed that miRNAs deregulation correlates and is involved with the initiation and progression of human tumors. Cervical cancer (CC) displays notably increased or decreased expression of a large number of cellular oncogenic or tumor suppressive miRNAs, respectively. However, understanding the potential role of miRNAs in CC is still limited. In CC, the high-risk human papillomaviruses (HR-HPVs) infection can affect the miRNAs expression through oncoprotein E6 and E7 that contribute to viral pathogenesis, although other viral proteins might also be involved. This deregulation in the miRNAs expression has an important role in the hallmarks of CC. Interestingly, the miRNA expression profile in CC can discriminate between normal and tumor tissue and the extraordinary stability of miRNAs makes it suitable to serve as diagnostic and prognostic biomarkers of cancer. In this review, we will summarize the role of the HR-HPVs in miRNA expression, the role of miRNAs in the hallmarks of CC, and the use of miRNAs as potential prognostic biomarkers in CC.

## 1. Introduction

MicroRNAs (miRNAs) are noncoding regulatory RNAs of 18–25 nucleotides in size that are derived from coding or noncoding sequences [1]. Many miRNAs are tissue- or differentiation-specific, and their temporal or short-lived expression modulates gene expression at the posttranscriptional level by base-pairing with complementary nucleotide sequences of target mRNAs [1]. Similarly to genes that encode for mRNAs, specific miRNAs were classified as oncogenes (oncomirs) or tumor suppressor genes based on their expression patterns in tumors [1, 2]. Likewise there is sufficient evidence that some miRNAs possess a tumor suppressive/proapoptotic role while others have antiapoptotic/proliferation promoting roles in the cell [3–5]. Moreover, miRNA signatures have indicated that aberrant (increased or decreased) miRNA expression is common in most human tumors [6] and genome wide miRNAs analyses indicate that approximately 50% of miRNA genes are located at fragile sites (FRAs), as well as in minimal regions of loss of heterozygosity or minimal regions of amplification, which are associated with cancers [2, 7, 8].

The first report documenting abnormalities in miRNA expression in tumor samples was on B-cell chronic lymphocytic leukemia (B-CLL), where miR-15 and miR-16 are frequently deleted and downregulated in B-CLL patients [9]. Subsequently, many other studies on different cancer types have shown deregulation in miRNAs expression. Thus, one major mechanism that underlies the roles of miRNAs in cancer development could be deregulated miRNA expression as compared with normal cells [10].

Studies have identified chromosomal alterations, gene expression changes, and aberrant promoter methylation associated with cervical cancer (CC) [10], but little is known about the specific role of miRNAs. However, it has been experimentally verified that some miRNAs play an important role in the hallmarks of CC such as cell cycle progression/proliferation, apoptosis, angiogenesis, immune response, invasion, and metastasis. These processes are commonly deregulated in cancer, indicating the involvement of miRNAs in CC.

Whereas the importance of miRNAs in human carcinogenesis is becoming increasingly recognized, the understanding of the potential role of miRNAs in cervical carcinogenesis is still limited but postulated by a number of studies. Furthermore, it has been shown that high-risk human papillomaviruses (HR-HPVs), despite producing no viral miRNAs, are responsible for the upregulation of oncogenic or downregulation of tumor suppressive miRNAs and these observations could shed more light on HR-HPV-induced oncogenesis [11, 12]. In addition, it has shown a differential expression of mature miRNAs during the consecutive stages of cervical squamous cell carcinoma (SCC) development [10]. These observations suggest that many aberrantly expressed miRNAs in CC could serve as diagnostic and prognostic biomarkers. In this review, we will summarize the role of miRNAs in the hallmarks of CC, the role of the HR-HPV on miRNAs expression, as well as the possibility of using miRNAs as potential prognostic biomarkers or therapeutic agents in the cervical carcinogenesis.

## 2. miRNAs Expression and Their Regulation Mechanisms in Cervical Cancer

Nowadays, it is well known that miRNAs can be upregulated or downregulated in various human cancers. Overexpressed miRNAs in cancer may function as oncogenes and promote cancer development by negatively regulating tumor suppressor genes and/or genes that positively control cell differentiation or apoptosis, whereas underexpressed miRNAs in cancer function as tumor suppressor genes and may inhibit cancers by regulating oncogenes and/or genes that control cell differentiation or apoptosis [2, 13–15]. Upregulation of miRNAs in human cancers can result from amplification, deregulation of a transcription factor, or demethylation of CpG islands in the promoter regions of the corresponding genes. MiRNAs acting as tumor suppressors can be downregulated in cancer by deletions, epigenetic silencing, or loss of transcription factor expression [15].

### 2.1. Downregulated Tumor Suppressor miRNAs in Cervical Cancer

**Figure 1:**
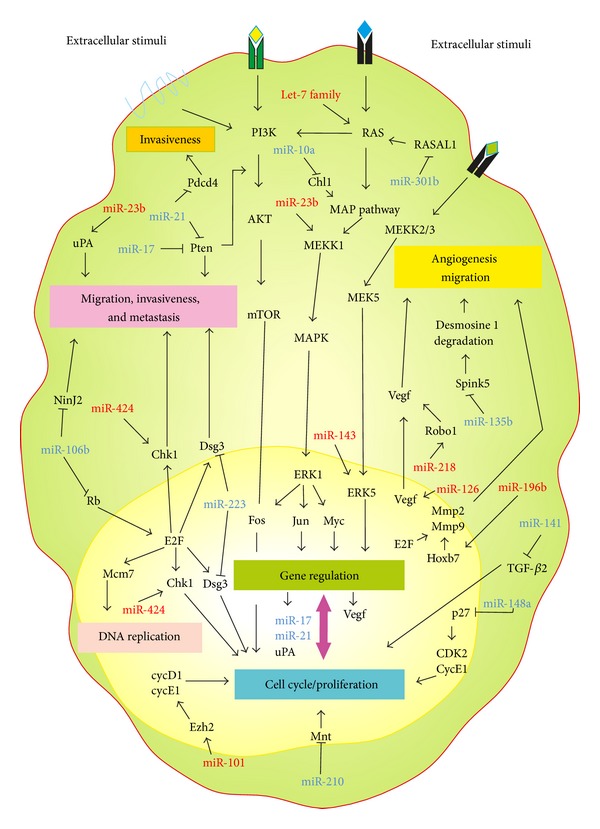
A schematic model for the putative roles of the miRNAs in different cellular processes such as cell cycle, apoptosis, proliferation, invasiveness, migration, metastasis, and angiogenesis in cervical cancer. Bar-headed lines represent inhibition, whereas arrows indicate stimulation. miRNAs in red represent downregulated (tumor suppressor) expression and miRNAs in blue represent upregulated (oncogenic) expression in cervical cancer. Chl1: cell adhesion molecule with homology to L1; CycE1: cyclin E1; Dsg3: desmoglein 3; Mnt: MAX dimerization protein; NinJ2: ninjurin 2; Pdcd4: programmed cell death 4; RASAL1: RAS protein activator like 1; SpinK5: serine peptidase inhibitor Kazal type 5; and VEGF: vascular endothelial growth factor.

Several miRNAs are downregulated and behave as tumor suppressors in CC and cell lines derived from this carcinoma (Table 1). For example, the let-7 family was the first group of oncomirs (miRNAs related with cancer) identified. These negatively regulate the expression of oncogenes, specifically the *RAS* genes. The overexpression of the *RAS *oncogenes has been observed in CC [16], and the expression of let-7 is found significantly reduced in CC cell lines (Table 1) [17, 18]. RAS proteins are membrane proteins that regulate cell growth and differentiation through NF*κ*B, PKB/AKT, and MAP kinase signaling. *In vitro *experiments showed that let-7 was able to inhibit cell proliferation through *RAS* downregulation, inferring that this miRNA may function as a tumor suppressor in this context [19]. Thus, in cervical cancer let-7 miRNA through the RAS protein expression indirectly alters the cell proliferation by the downstream MAP kinase signaling cascade (Figure 1).

Other downregulated miRNAs in CC have also been associated with tumor angiogenesis. For example, miR-23b normally inhibits a cohort of prometastatic genes or oncogenes, including *FZD7*, *MAP3K1*, *PAK2*, *TGF*β*R2*, *RRAS2*, or *uPA* [20]. Moreover, it has been reported that miR-23b is downregulated in CC lines [18], which leads to increased expression of MAP3K1 [20, 21], which has been found overexpressed in HR-HPV-positive SCC [22]. These reports suggest that miR-23b downregulation might have a role not only in CC angiogenesis but also in metastasis (Table 1 and Figure 1).

The miR-101 plays a significant role in cell proliferation, migration, and angiogenesis through the inhibition of enhancer of zeste homolog 2 (EZH2) (Figure 1) [23]. In prostate cancer, there is an increase in the expression of EZH2, which appears to be due to decreased expression of miR-101 [24]. EZH2 is a histone methyl transferase that is highly expressed in several cancers and associated with tumor cell proliferation, invasion, and metastasis [25, 26]. In CC tissue, the EZH2 expression rate is greater than that in normal cervical tissue both in mRNA and protein level [25]. A previous report showed that the EZH2 oncogene induces the inactivation of tumor suppressor genes (MYT1, WNT1, KCNA1, and CNR1) in HeLa nuclear extracts [27] and it has been also found in stimulating expression of a series of proliferative genes, including several cyclin genes, such as *Cyclin D1* and *Cyclin E*, that are required for the proliferation of CC cells [28]. Wang et al. showed that the expression of miR-101 is frequently reduced in CC (Table 1) [11], which suggests that activation of miR-101 by novel approaches could become a promising target for the inhibition of CC cell proliferation.

It was previously reported that miR-126 is a suppressor of breast cancer metastasis [29]. Li et al. and Martinez et al. found that miR-126 is downregulated in CC (Table 1) [17, 30]. Interestingly, this miRNA can inhibit the overexpression of VEGF in tumor cells [31], blocking endothelial cell division, proliferation, and migration [31]. The direct correlation observed between VEGF overexpression and the process of angiogenesis in CC [22, 32] suggests that miR-126 is a potential tumor suppressor gene that may play a critical role in the process of CC angiogenesis (Figure 1).

Several studies have demonstrated that the expression levels of miR-143 and miR-145 are significantly lower in CC as compared with their normal counterparts (Table 1) [11, 18, 30]. Wang et al. showed that these miRNAs suppress cervical (HeLa) cancer cell growth [11], suggesting that downregulation of miR-143 and miR-145 might lead to cell proliferation in CC (Figure 1). It is important to note that the only experimentally verified target for miR-143, *ERK5* (also known as MAPK7), is known to promote cell growth and proliferation in response to tyrosine kinase signaling [33]. The ERK5 role in CC cells and its regulation by miR-143 are subject of further investigation.

It was recently found that enforced expression of miR-196b reduced *in vitro *invasion and *in vivo *spontaneous metastasis of breast cancer cells, indicating that this miRNA plays a tumor suppressor function [34]. In CC lines, miR-196b is downregulated (Table 1) [18] and it has been observed that downexpression of miR-196b in human CC likely promotes tumor cell proliferation via increased activity of the HOXB7 transcription factor, which in turn can upregulate several prosurvival pathways, ultimately leading to tumor progression (Figure 1) [35, 36].

Previous publications reported that miR-218 was downregulated in several cancers such as bladder, lung, and oral cancer [37–39]. It was demonstrated that miR-218 could inhibit tumor invasion and metastasis by targeting Robo1 [40], which is an oncogenic transmembrane receptor of the immunoglobulin family that upon binding to its ligand, Slit2, cooperates with the Abl kinase to induce cell migration [41, 42]. Several studies have shown that the expression of miR-218 is frequently reduced in CC (Table 1) [11, 17, 30, 43] and a recent study reported that downregulation of miR-218 had a strong relationship with later stages of SCC, cervical adenocarcinoma, and lymphatic node metastasis (Figure 1) [44], confirming that miR-218 can act as a tumor suppressor miRNA in CC.

miR-424 is another interesting miRNA that can act as a potential tumor suppressor [45, 46] in CC; it was downregulated in both CC tissues and cells derived from these tumors employing microarray assays (Table 1) [11, 17]. The restoration of miR-424 expression in the human cervical cancer cell lines SiHa and CaSki remarkably affected several cell biological behaviors, including the inhibition of cell growth, by both enhancing apoptosis and blocking G1/S transition, and the suppression of cell migration and invasion [47]. Interestingly, overexpression of miR-424 inhibits the expression of protein checkpoint kinase 1 (CHK1) and the active phosphorylated form of CHK1 (pCHK1) [47]. CHK1 has for long been known as a key player in determining cellular responses to DNA damage and governing G1/S, S, and G2/M phase checkpoints [48]. Moreover, overexpression of CHK1 and pCHK1 is frequently observed in CC tissues and both proteins are positively correlated with the increased progression and metastatic spread of the disease [47]. On the other hand, knockdown of CHK1 expression mimic overexpression of suppressive miR-424 on CC cells, suggesting that downregulation of miR-424 contributes to the progression of CC at least partly via upregulation of CHK1 expression (Figure 1).

miR-195 is underexpressed in integrated HPV-16 cervical cell lines compared to the normal cervix; however, its regulation mechanisms and its targets are still not known.

### 2.2. Upregulated Oncogenic miRNAs in Cervical Cancer

Several miRNAs are overexpressed in CC, which suggests that these miRNAs may play an oncogenic role (Table 1). A first example are the members of the miR-10 family that have been reported to be upregulated in several cancers [7]. miR-10a was found to be overexpressed in colon cancer [49] and CC [50]. Recently, it was reported that Close Homolog of L1(CHL1), a type I transmembrane protein involved in cell adhesion, is a direct target of miR-10a and is negatively regulated at the mRNA and protein levels [51]. It was demonstrated that CHL1 is poorly expressed in CC tissue specimens, compared to the nontumor tissue specimens; it is also low expressed in HeLa and C33A cells (Table 1) [51]. Long et al. presume that, in CC cells, low expression of CHL1 leads to activation of the MAPK and PAK pathways, which affects downstream molecules contributing to cell growth, migration, and invasion [51]. These data demonstrate that the upregulation of miR-10a expression observed in CC tissues could have an important role in cell growth, migration, and invasion by targeting CHL1 mRNA in CC cells (Figure 1).

miR-21 is a highly overexpressed miRNA and considered oncogenic in numerous cancers [52, 53]. miR-21 downregulates the tumor suppressor *PTEN* in non-small cell lung cancer [52] and in hepatocellular cancer, in which PTEN downregulation leads to overexpression of matrix metalloproteinases MMP2 and MMP9, that promotes cellular migration and invasion [54]. These MMPs were found to be overexpressed in CC and have been associated with invasive features of cancers [55, 56]. miR-21 also targets the tumor suppressor genes tropomyosin 1 (TPM1), programmed cell death 4 (PDCD4), and Maspin; the latter two have been implicated in the suppression of tumor invasion and metastasis [57, 58]. PDCD4 has been observed downregulated in CC [59]; it seems that miR-21 acts directly on the *PDCD4* mRNA in HeLa cells, increasing cellular proliferation [60], suggesting that miR-21 may play an oncogenic role in CC invasion and metastasis (Figure 1).

It has been reported that miR-106b could be an oncogene in cancer, in part because it can promote breast cancer invasion and metastasis by targeting two important tumor suppressors: *BRMS1* and *RB* [61]. miR-106b is overexpressed in squamous cell carcinoma of the cervix (Table 1) [17] and is part of a cluster of miRNAs, along with miR-93 and miR-25, which are located within an intron of the minichromosome maintenance 7 (*MCM7*) gene. *MCM7*, a target of the E2F1 transcription factor, is a marker for CC and may be induced by both HPV16 E6 and E7 [62, 63]. Additionally, miR-106b may target ninjurin 2 (NINJ2), first cell surface adhesion molecule identified on neural cells [64], which is also downregulated in CC [65]. Reduction of NINJ2, via overexpression of miR-106b, could contribute to loss of adhesion and increased migration of CC cells (Figure 1).

miR-135b is overexpressed in colorectal cancer [66] and in CC [43] (Table 1). A potential target of miR-135b is the serine protease inhibitor Kazal type 5 (Spink5), which is downregulated in CC [65]. SPINK5 deficiency causes unregulated epidermal protease activity and degradation of desmoglein 1, which leads to inefficient stratum corneum adhesion and a resultant loss of skin barrier function [67]. Overexpression of miR-135b and loss of SPINK5 and desmoglein 1 could decrease cellular adhesion, increase proliferation, and change the epithelial architecture of the cervix and promote a tumorigenic phenotype (Figure 1).

In numerous cancers, including CC, miR-141 is also overexpressed (Table 1) [43, 68–70]. Recently, it was shown that miR-141 downregulates the expression of TGF*β*2 [71] which shows reduced expression in CC [72]. This suggests that the overexpression of miR-141 in the cervical epithelium could therefore contribute to cervical tumorigenesis (Figure 1).

miR-146a positively contributes to cell growth and survival, processes mainly related to cancer development and progression [73]. Recently, Wang et al. reported an increase in miR-146a expression in CC; they further observed that miR-146a promotes cell proliferation in CC cell lines [11] (Table 1). This suggests that miR-146a functions as a growth factor in CC. However, further investigations are needed to elucidate the molecular mechanism of miR-146a in CC.

Cell proliferation and cell cycle progression is promoted by miR-148a overexpression through p27 mRNA degradation, a CDK inhibitor [74]. Particularly interesting is the report of miR-148a overexpression (Table 1) [50] and p27 downregulation in CC tissues [75]. This suggests that miR-148a might promote cervical cell proliferation and CC by repressing p27 expression (Figure 1).

miR-210 is located within the intron of a noncoding gene on human chromosome 11 and it is highly expressed in CC (Table 1) [30]. Recently, it has been reported that miR-210 directly targets MNT mRNA [76]. MNT is a MAX-interacting transcriptional repressor that functions as a *c-MYC* antagonist. Inhibition of MNT expression promotes c-MYC activity and cell cycle progression in transformed cells such as colon cancer cells and CC cells (Figure 1) [77]. Since it is well documented that MYC promotes cell proliferation in cervical cancer and in squamous intraepithelial lesions of the uterine cervix [78], it is possible that miR-210 is involved in cervical carcinogenesis through the promotion of MYC overexpression.

It was found that miR-214 is highly expressed in ovarian tumors and functions as an oncogene that inhibits the tumor suppressor *PTEN*, leading to activation of the AKT pathway [79]; it is also overexpressed in CC tissue (Table 1) [43, 80]. Interestingly, tumor progression in the cervical epithelium is accompanied by loss of PTEN protein expression and elevated PI3-kinase activity that contribute to growth promotion through the AKT pathway [81, 82]. Therefore, these data indicate that miR-214 could be a causal factor for the downregulation of PTEN in CC, leading to tumor progression and cell survival via the upregulation of AKT (Figure 1) (see also* miR-21*).

Overexpression of miR-223 was demonstrated in the myeloid cells of the bone marrow [83]. MiR-223 may target desmoglein 3 (*DSG3*), which is downregulated in cervical intraepithelial neoplasia grade 3 (CIN 3) and CC [65]. Desmoglein 3, a desmosomal adhesion molecule, participates in intercellular links via desmosome-intermediate filament complexes and helps to maintain tissue integrity. Reduced expression of desmoglein 3 increases the colony-formation efficiency and proliferative potential of primary keratinocytes [84]. Wang et al. and McBee et al. reported overexpression of miR-223 in cell lines derived from CC and in CC tissues [11, 43] (Table 1). These observations suggest that miR-223 may reduce cellular adhesion and promote proliferation of cancer cells as previously determined for miR-135b (Figure 1).

miR-301b is overexpressed in colon cancer tissue [66] and CC (Table 1) [43]. This miRNA may target the tumor suppressor RASAL1, which inhibits the RAS protein signal transduction [43, 85]. RASAL1 expression is lost in cancers, including CC, which promotes activation of the RAS oncogene [65, 85]. So, overexpression of miR-301b could potentially increase RAS activation and promote CC via the downregulation of RASAL1 (Figure 1).

Importantly, the maintenance of homeostasis in mammalian systems requires a tight control in all cellular processes and the upregulation or downregulation in the miRNAs expression plays an important role in the hallmarks of CC (Table 2).

## 3. High-Risk Human Papillomaviruses (HR-HPVs) Modify the miRNAs Expression in Cervical Cancer

Human papillomaviruses are a group of small DNA viruses that contain a small, double-stranded circular genome of ~8 kb and encode six viral early proteins (E6, E7, E1, E2, E4, and E5) [86]. Certain types of HR-HPVs, such as HPV16 and HPV18, have been recognized as causative agents of CC and their infections are associated with ~ 74% of squamous cell carcinomas and 78%–81% of adenocarcinomas [87, 88]. Therefore, it is conceivable that oncogenic HPV infection causes aberrant expression of cellular miRNAs. Recent studies indicate that multiple miRNAs have altered expression in HR-HPV containing CC cells as compared with HPV-negative CC cells or normal cervical tissues [30, 89].

Calin et al. reported that one cluster of FRAs at chromosome 17q23 contains three HPV16 integration events and four miRNA genes (miR-21, miR-301, miR-142s, and miR-142as); they conclude that these miRNAs are possible targets of such viral integration that may lead to deregulation of miRNAs expression [8]. It is well know that a significant number of miRNAs are located in FRAs and that such sites are preferential targets for HPV16 integrations, sister chromatid exchange, translocation, deletion, and amplification in cervical tumors [8, 90]. In addition, HPV16 positive CaSki and SiHa cell lines and HPV-positive SCC present miR-21 overexpression as compared with normal uninfected tissues [91] which could indicate that miR-21 upregulation might be the result of HPV integration. However, a direct comparison of the miRNA expression profiles in integrated versus episomal HPV16 cell lines did not reveal any significant difference [30]. This observation supports the idea that only the infection not the integration by HR-HPV is sufficient to modify the miRNAs expression.

miRNAs expression profiles by microarray analysis have shown that the miRNAs most highly expressed in normal cervix were miR-145, miR-26a, miR-99a, let-7a, miR-143, let-7b, let-7c, miR-125b, miR-126, and miR-195, in that order [11, 18, 30, 31]. Importantly, some miRNAs are similarly deregulated while others are not in HPV16 and HPV18 positive cell lines [30].

- HPV16 positive SiHa and CaSki cell lines have shown an overexpression of miR-182, miR-183, and miR-210. In contrast, miR-1, miR-126, miR-133b, miR-143, miR-145, miR-195, miR-214, miR-368, miR-451, and miR-7029 are underexpressed in these cell lines as compared with normal cervical tissue.
- HPV18 positive HeLa cell line has overexpression of miR-182 and miR-183, while miR-1, miR-133b, miR-143, miR-145, miR-214, miR-368, miR-451, and miR-7029 are underexpressed in this cell line as compared with normal cervical tissue.

Thus, miR-182 and miR-183 are overexpressed and miR-1, miR-133b, miR-143, miR-145, miR-214, miR-368, miR-451, and miR-7029 are underexpressed in both cell lines containing integrated HPV16 or HPV18 DNA [30]. Interestingly, it has been shown that also miR-218 is underexpressed in the cell lines containing integrated HPV16 DNA as compared to both the normal cervix and HPV-negative C-33A cell line. So, this could suggest that miR-218 may be specifically affected by the presence of HPV16 [30]. These data show that the infection by HR-HPVs plays an important role in the deregulation of the miRNAs expression in CC.

### 3.1. Modulation of Cellular MicroRNAs Expression by HPV E6 and/or E7 Oncoproteins in Cervical Cancer

As previously mentioned, overexpression of oncogenic and underexpression of tumor suppressive miRNAs in human CC are frequently associated with HR-HPV. This altered expression of some miRNAs most likely occurs through the viral oncoproteins.

#### 3.1.1. Downregulated MicroRNAs by E6

**Figure 2:**
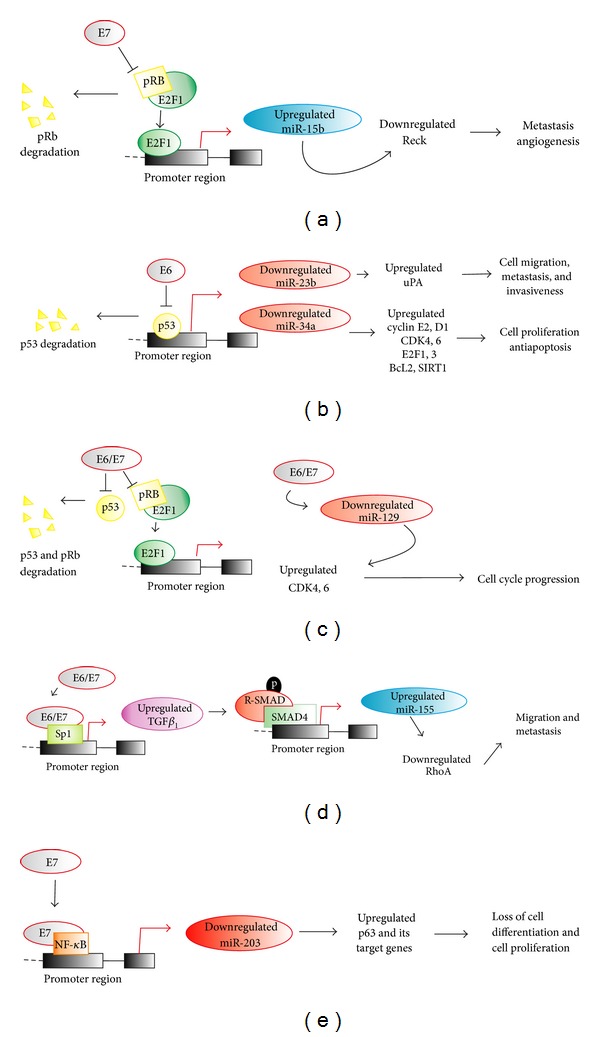
HR-HPV oncoproteins in the regulation of microRNAs and cellular processes affected in cervical cancer. RECK; reversion-inducing cysteine-rich protein with Kazal motifs.

HR-HPV E6 oncoprotein through the degradation of p53 [92] is capable of regulating the expression of numerous coding and noncoding genes including miRNAs [14]. In this regard, HR-HPV16 E6 oncoprotein through the degradation of p53 was found to decrease the expression of miR-23b, which leads to the increase in the expression of urokinase-type plasminogen activator (uPA), and thus induces the migration of human cervical carcinoma SiHa and CaSki cells [93]. This mechanism is supported by several evidences: knockdown of HPV16 E6 by HPV16 E6 siRNA transfection was confirmed to increase miR-23b expression and decrease uPA expression in SiHa and CaSki cells; the 3′-untranslated region of uPA mRNA has binding sites for miR-23b and negatively regulates uPA expression; the tumor suppressor p53 has a consensus binding site and activates the promoter region of miR-23b [93]. Interestingly, miR-23b is often downregulated in HR-HPV-associated cervical cancer and the upregulation of uPA induced by reduced expression of miR-23b is known to play a key role not only in cell migration but also in metastasis and invasiveness [94] and is considered a prognostic indicator of CC (Figure 2(b)) [95].

Similarly, miR-34a has also been identified as a direct transcriptional target of p53. The transactivation of miR-34a expression is triggered by the binding of p53 to a consensus p53 binding site identified in the miR-34a promoter region [96, 97]. Since HPV E6 oncoprotein destabilizes p53 during virus infection, one may assume that miR-34a expression is downregulated in most CC tissues with oncogenic HPV infection. Interestingly, Wang et al. showed that, at all stages of pathogenesis induced by the HR-HPV types, the E6 oncoprotein expressed from HPV16 or HPV18 inhibits the expression of tumor suppressive miR-34a by destabilization of p53, resulting in cell proliferation (Figure 2(b)) [89]. It has been documented that transfection of miR-34a induces cell cycle arrest in immortalized mouse cells and in human tumor cell lines and sensitizes to apoptosis in response to genotoxic stress [14, 96], which is consistent with the observed ability of miR-34a to downregulate genes promoting cell cycle including *Cyclin E2*, *Cyclin D1*, *CDK4*, *CDK6*, *E2F1*, *E2F3*, *E2F5*, and *SIRT1* [14, 98]. In addition, miR34a upregulates *p18Ink4c*, a* CDK4* and *CDK6* inhibitor of the *INK4* family [99], and downregulates the antiapoptotic *Bcl-2* mRNA.

The miR-218 expression is downregulated in several cancers and is frequently deleted in ovarian, breast, and melanoma cancers [1]. Although miR-218 is not defined as a transcriptional target of p53, this miRNA is underexpressed in the presence of the E6 oncoprotein [30]. miR-218 is specifically underexpressed in cervical cell lines, cervical lesions, and cancer tissues containing integrated HPV16 DNA compared to the normal cervix. Moreover, Martinez et al. revealed that exogenous expression of the HPV16 E6 oncogene reduced miR-218 expression, and, conversely, RNA interference of E6/E7 oncogenes in an HPV16 positive cell line increased miR-218 expression. Exogenous expression of miR-218 in HPV16 positive cell lines decreases LAMB3transcript and protein levels, a target of miR-218 which increases cell migration and tumorigenicity [30]. These results suggest that the downregulation of miR-218 by E6 and the consequent overexpression of LAMB3 may contribute to cervical tumorigenesis [30].

#### 3.1.2. Upregulated MicroRNAs by E7

The HR-HPV E7 oncoprotein upregulates the expression of the oncogenic miR-15b in CC [100]. In fact, high expression of miR-15b is observed in CC (Table 1) [17] that correlates with increased *CCNA2*, *CCNB1*, *CCNB2*, and *MCM7* expression, E2F-induced genes that favor proliferation [100]. It is known that E7-mediated degradation of pRB releases E2F from the pRB-E2F complex to act as transcriptional factor [101]. Thereby, members of the E2F family of transcription factors are used by the HR-HPV to take control of proliferation in the infected cell [17, 100, 102]. Myklebust et al., Ofir et al., and Bueno et al. established that expression of miR-15a, miR-15b, and miR-16 is positively regulated by E2F1 and E2F3 [100, 103, 104], suggesting a mechanism for the upregulation of these miRNAs by the E7 oncoprotein.

On the other hand, it was observed that the expression of the E7 oncoprotein results in decreased levels of RECK reversion-inducing cysteine-rich protein with Kazal motifs (RECK) [105], a membrane bound protein that can exert inhibitory effects on the transcription, synthesis activity of MMPs [106, 107]. It has been observed that miR-15b targeting to Reck mRNA (Figure 2(a)), which is downregulated during cervical cancer initiation/progression [105]. These data suggest that miR-15b may play an important role in invasion/metastasis/angiogenesis by targeting RECK.

#### 3.1.3. Downregulated MicroRNAs by E7

miR-203 is a critical molecule for inducing the transition of keratinocytes from a proliferative state in undifferentiated basal cells to a nonproliferative status in differentiated suprabasal cells [108]. This important miRNA is negatively regulated by the HR-HPVE7 oncoprotein causing a differentiation block [109]. In keratinocytes, the p63 family of transcription factors was identified as a primary target of miR-203 action, with ΔNp63 being the primary member expressed in epithelia and negatively regulated by miR-203 [108, 110].

ΔNp63 is expressed at high levels in proliferating undifferentiated basal keratinocytes but is downregulated in normal differentiated nonproliferating cells [111]. These changes in the ΔNp63 expression suggest that this protein is a regulator of the balance between epithelial proliferation and differentiation [111]. Moreover, the HR-HPVE7 oncoprotein is the primary viral factor responsible for blocking expression of miR-203, probably by negative regulation of the transcription factor NF*κ*B which has binding sites in the miR-203 promoter region (Figure 2(e)) [109, 112]. Otherwise, several downstream targets of p63 were increased in E7-expressing cells, and their levels were inversely correlated with amounts of miR-203. The expression of miR-203 is also significantly reduced in cells where p53 function is compromised or after the degradation of p53 induced by E6, thereby providing more evidence as to how HPV infection could lead to deregulation of proliferation and differentiation in keratinocytes during the development of CC [113].

#### 3.1.4. Upregulated or Downregulated MicroRNAs by Both E6 and E7

miR-129 is considered a candidate miRNA with potential tumor suppressor activity. This miRNA may suppress cell growth; but its expression levels are much lower in tumor cell lines or tumors derived from neural, gastric, colorectal, and cervical tissue than in their respective normal tissues [17, 50, 114–117]. CDK6 is a direct target of miR129 in CC, so downregulation of miR-129 results in upregulation of CDK6 and cell cycle deregulation in this carcinoma. Li et al. found that CDK6 was positively correlated with E6/E7 mRNA expression [17]. Furthermore, overexpression of CDK6, CDK4, and cyclin D1 is present in all cases of CC [78], which is believed to be a primary event that facilitates G1-S cell cycle progression. This action requires cyclin D1 binding to CDK4 and CDK6, which phosphorylate and inactivate RB1, leading to the release of E2F transcription factors and the subsequent progression of the cell into the S phase [17]. Data demonstrate that downregulation of miR129 and upregulation of CDK6 by either E6 or E7 oncoprotein cooperate with cyclin D1 to further promote cell cycle progression in CC (Figure 2(c)).

On the other hand, the miR-155 oncogene is an important miRNA upregulated by the TGF*β*/Smad4 pathway. This miRNA contributes to the TGF*β* induced epithelial-mesenchymal transition (EMT) and cell migration and invasion by targeting RhoA (ARHGAP12) [118]. RhoA has a positive or negative impact in cellular processes, including the promotion of cellular adhesion and polarity and decreased motility, and is an important modulator of cell junction formation and stability [119]. Previously, it was reported that RhoA is negatively regulated by miR-155 contributing to tight junction disruption. E6 and E7 oncoproteins from HPV16 induce activation of TGF*β*_1_ [120], which has been implicated in metastatic CC [121]. Otherwise, RhoA has been reported to be downregulated (−2.7-fold change) in CC, suggesting negative regulation by E6 and E7 oncoproteins through TGF*β*_1_ [122]. Additionally, miR-155 has been found to be overexpressed in invasive breast cancer [49, 123] and CC (Table 1) [17]. These data suggest that miR-155 may play an important role in cell migration and invasion by targeting RhoA and indicate that this *oncomir* is a potential therapeutic target for cervical cancer (Figure 2(d)).

### 3.2. The E5 Oncoprotein Also Regulates the Expression of miRNAs

Although it has been shown that the E6 and E7 viral oncoproteins play important roles in the expression of miRNAs, this does not exclude the possibility that the expression of many other miRNAs in CC could be deregulated due to the HR-HPVE5 oncoprotein or due to epigenetic control unrelated to viral gene expression. The E6 and E7 oncoproteins are well known to induce tumorigenicity and the E5 oncoprotein may enhance the effect of E6 and E7 oncogenes in CC [124, 125]. Recently, it was suggested that E5 alone might have high oncogenic potential, because E5 transgenic mice were shown to develop CC after prolonged estrogen treatment [124] as was previously shown for HR-HPV E7 transgenic mice [126]. It has been observed that genes implicated in cell motility and cell adhesion are affected by E5 expression, which suggests that E5 is highly implicated in the carcinogenic process [125].

However, the specific effect of the E5 oncogene on genome-wide expression of known human miRNAs has been least studied so far. The effect of HPV16E5 on the expression of host miRNAs was studied in uninduced HaCaT cells as well as after 24, 48, and 72 hours of E5 induction (HaCaT-E5) under the control of a dexamethasone inducible promoter [127]. miRNAs microarray analysis showed that, in the presence of E5, miR-324-5p was constantly downregulated and miR-146a was constantly found upregulated in HaCaT-E5 cells at all time points examined [127]. Interestingly, a strong upregulation of N-cadherin, which is a predicted target of miR-324-5p, and downregulation of PDZD2, which is target of miR-146a, have been shown [127]. The cadherins not only establish adherens junctions but also regulate molecular polarity during migration. N-cadherin is required for microtubule organizing centre orientation and directional migration of smooth muscle cells [128]. In cervical dysplasia, N-cadherin is expressed at cellular junctions throughout the epithelium, whereas the expression in normal tissue is restricted to the bottom layers of the epithelium [127]. Intriguingly, upregulation of mesenchymal markers such as N-cadherin is seen in epithelialmesenchymal transition (EMT), a crucial process activated in cancer and generating cells with stem cell properties [129]. PDZD2 encodes a PDZ domain protein, which is a component of the cellular adhesion complexes, including tight and adherent junctions. Additionally to the posttranscriptional negative regulation of PDZD2 by miR-146a, HR-HPVE6 may potentially interact with this PDZ domain containing protein and, after E6AP ubiquitin ligase recruitment, induce its degradation [130].

Although Melar-New and Laimins previously reported that miR-203 is constantly repressed by the HR-HPV E7 proteins with subsequent upregulation of ΔNp63 [109], miR-203 downregulation and modest upregulation of p63 were also observed at late time points (96 h) in HaCaT-E5 cells [127], suggesting that the oncogenic roles of both the E7 and the E5 proteins are mediated by miR-203, but the regulation by the HPVE7 oncoprotein could be more important than the regulation by the E5 oncoprotein. These observations indicate that HR-HPV infection and subsequent transformation take place through complex patterns of gene expression in the host cells, regulated in part by the E5 oncoprotein and related miRNAs.

## 4. miRNAs in the Prognostic of Cervical Cancer

The miRNAs in mammalian cells have an average half-life of around 5 days, 10 times more than that of regular mRNAs [131]. These small RNAs are resistant to degradation by RNase A [132], high temperature, extreme pH, and freeze-and-thaw cycles [133]. These characteristics of miRNAs make them suitable to serve as prognostic biomarkers of diseases. The miRNA expression profile can discriminate between normal tissue and tumor tissue and this differential expression can also be used as a prognostic marker for clinical aggressiveness of human cancer [134]. For example, Calin et al., in chronic lymphocytic leukemia, reported a miRNA expression signature composed of 13 mature miRNAs that were associated with prognostic factors and disease progression [135]. Roldo et al. revealed in pancreatic endocrine tumors and pancreatic acinar cell carcinomas that the overexpression of miR-21 was strongly associated with a high Ki67 proliferation index and the presence of liver metastasis [136]. Blenkiron et al. reported that analysis of miRNA expression could distinguish between mammary invasive ductal carcinoma and mammary medullary carcinoma [137]. In lung squamous cell carcinoma, miR-155, let-7, and miR-146a are of prognostic use [138]. Given the significant association of miRNA expression profiles with carcinogenesis, it is likely that a selected set of miRNAs could be used to build a predictive model for CC prognosis. Thus, Hu et al. proposed that the level of tumors suppressor miR-200a and miR-9 could predict CC patient survival. Both miRNAs were significantly associated with disease outcome and local recurrence-free survival, as well as with independent prognostic value from clinicopathologic features [139]. The authors suggest that miR-200a and miR-9 could play important regulatory roles in CC control because miR-200a is likely to decrease the metastatic potential of CC cells by coordinate suppression of multiple genes inducing cell motility; miR-9 could potentially be involved in tumor control blocking the high metabolic rate important for the rapid proliferation of CC cells [140]. Furthermore, high levels of the oncogenic miR-210 were associated with disease recurrence and short overall survival in head and neck squamous cell carcinoma and this miRNA is also upregulated in CC [30, 141]. Several authors have reported that increased miR-127 and miR-21 expression were associated with cervical carcinogenesis and lymph node metastasis [90, 134]. In contrast, low levels of miR-218 and miR-424 have also shown to have a good prognostic role in CC [44, 47]. This shows that many aberrantly expressed miRNAs in CC could serve as prognostic biomarkers (Table 1). Furthermore, the development of a biomarker panel may be useful in determining which patients with cervical dysplasia are likely to progress to more invasive disease, and it may also be a useful prognostic indicator in patients with invasive cancer. However, more studies are necessary to determine the diagnostic and prognostic implications of many differentially expressed miRNAs in CC.

## 5. Conclusion

The above studies show that miRNAs are emerging as contenders for future biomarkers of cervical cancer. The fact that differing levels of miRNAs have been detected between cervical cancer cases belonging to specific histopathological groups and healthy controls certainly promotes these molecules as potential biomarkers. Moreover, these studies provide evidence that there is interplay between viral oncoproteins and miRNAs that could be used as early biomarkers in CC and potentially useful to determine disease prognosis. However, future studies are needed to dissect the function, transcriptional targets, and the mechanisms by which many miRNAs regulate the cellular events within both normal and cervical cancer tissue that could give better information on their role as biomarkers, as well as in prognosis and therapy of cervical cancer.

## Figures and Tables

**Table 1:** MicroRNAs expression in cervical cancer.

Author	Downregulated	Upregulated	Materials and methods	Reference
Wang et al., 2008	miR-23b, miR-34a, miR-101, miR-143, miR145, miR-218, and miR-424,	miR-15a, miR146a, and miR-223	HPV16^+^, HPV18^+^, and HPV negative cervical cancer cell lines; cervical cancer versus age matched normal cervix samples; microarray platform, Northern blot	[11]

Li et al., 2011	Let-7b, miR-10b, miR-29a miR-99a, miR-125b, miR-126, miR-218, miR-375, and miR-424	miR-15b, miR-16, miR-17, miR-20a, miR-20b, miR-93, miR-106a, miR-155, and miR-224	Cervical fresh frozen tissues including SCC (*n* = 51), CIN2-3 (*n* = 51), and HR-HPV infected normal cervix with viral cytopathic effect (*n* = 21) and disease-free histologically normal specimens (*n* = 49); microarray platform, quantitative RT-PCR	[17]

Lui et al., 2007	let-7a-c, miR-23b, miR-143, and miR-196b	miR-21	Six cervical cancer cell lines and five normal cervical samples; cloning-based technique, Northern blot	[18]

Martinez et al., 2008	miR-126, miR-143, miR145, miR-195, miR-218, miR-368, and miR-497	miR-182, miR-183, and miR210	HPV16^+^, HPV18^+^, and HPV negative cervical cancer lines and cervical cancer tissue samples versus normal cervix samples; microarray platform, Northern blot, quantitative RT-PCR	[30]

Gardiner et al., 2011	miR-218 and miR-433	miR-21, miR-124, miR-135b, miR-141, miR-223, miR-301b, miR-449a, miR-449b, miR-517a, miR-517c, and miR-545	Six cervical cancer biopsies, three types of cervical dysplasia, and four normal cervical tissues; microarray platform, *quantitative *RT-PCR	[43]

Pereira et al., 2010	miR-26a, miR-29a miR-143, miR-145, miR-99a, miR-199a, miR-203, and miR-513	miR-10a, miR-132, miR-148a, miR196a, and miR-302b	Seven cases of high-grade SIL (CINII, *n* = 2, and CIN III, *n* = 5), 9 cases of low-grade SIL (CIN I), and 19 normal cervix samples; microarray platform, *quantitative * RT-PCR	[50]

Lee et al., 2008	miR-149 and miR203	miR9, miR127, miR-199b, miR-199s, and miR-214	Ten cervical cancer tissue samples versus ten normal cervix controls; *quantitative * RT-PCR	[80]

**Table 2:** miRNAs and the hallmarks of cervical cancer.

Hallmarks	Functions of miRNAs	miRNAs
Regulation of cell proliferation	Proproliferation	**miR-10a**, **miR-15b**, miR-17, miR-19a/b, **miR-21**, **miR-34a**, **miR-129**, miR-146a, miR-148a, **miR-196, miR-210**, miR-223, and miR-301b.
	Antiproliferation	**let-7**, miR23b, miR-26a, miR-101, miR-143, miR-145, miR-196b, 199a, **miR-203, **miR-424, and miR-519.

Regulation of apoptosis	Proapoptosis	**miR-34a**, miR-10b, miR-29a, miR-99a, and miR-195.
	Antiapoptosis	miR-17, **miR-21**, miR-149, miR-182, **miR-203**, miR-433, and miR-497.

Regulation of limitless replicative potential	Regulation of immortalization or senescence	miR-24 and miR-34a.

Regulation of angiogenesis	Proangiogenesis	let-7, miR-17, miR-20a, **miR-21**, miR-23b, miR-26a, miR29a, **miR-126**, miR133a, miR-183, miR-210, and miR-214.
	Antiangiogenesis	**miR-15b**,** miR-16**, miR-20a, miR-26a, miR-143, miR-145, miR-424, and miR497.

Regulation of immune responses	Escape from immunosurveillance	**miR-155**, miR-17, miR-23b, miR-26a, miR-29a, miR-17, miR-20a, miR-21, miR-93, and miR-106b.

Regulation of tumor invasion and metastasis	Prometastasis	**miR-10a**, miR-19a/b, **miR-21**, **miR-23b**, miR-106b, **miR135**,** miR-155**, and **miR-214**.
	Antimetastasis	let-7, miR-101, **miR-126**, miR-146a, miR-200, and **miR-218**.

Regulation of genomic instability	Promote genomic instability	**let-7**,miR-15, miR-16, miR-17, and miR20a.

Information obtained from *microRNA.org-Targets and Expression*. miRNAs in bold were experimentally validated in cervical cancer; underlined miRNAs were experimentally validated in other cancers.

## References

[1] Calin GA, Croce CM (2006). MicroRNA signatures in human cancers. *Nature Reviews Cancer*.

[2] Zhang B, Pan X, Cobb GP, Anderson TA (2007). MicroRNAs as oncogenes and tumor suppressors. *Developmental Biology*.

[3] Calin GA, Croce CM (2006). MicroRNA signatures in human cancers. *Nature Reviews Cancer*.

[4] Kent OA, Mendell JT (2006). A small piece in the cancer puzzle: microRNAs as tumor suppressors and oncogenes. *Oncogene*.

[5] Miska EA (2005). How microRNAs control cell division, differentiation and death. *Current Opinion in Genetics and Development*.

[6] Farazi TA, Spitzer JI, Morozov P, Tuschl T (2011). MiRNAs in human cancer. *Journal of Pathology*.

[7] Zhang L, Huang J, Yang N (2006). MicroRNAs exhibit high frequency genomic alterations in human cancer. *Proceedings of the National Academy of Sciences of the United States of America*.

[8] Calin GA, Sevignani C, Dumitru CD (2004). Human microRNA genes are frequently located at fragile sites and genomic regions involved in cancers. *Proceedings of the National Academy of Sciences of the United States of America*.

[9] Calin GA, Dumitru CD, Shimizu M (2002). Frequent deletions and down-regulation of micro-RNA genes miR15 and miR16 at 13q14 in chronic lymphocytic leukemia. *Proceedings of the National Academy of Sciences of the United States of America*.

[10] Wilting SM, Snijders PJF, Verlaat W (2012). Altered microRNA expression associated with chromosomal changes contributes to cervical carcinogenesis. *Oncogene*.

[11] Wang X, Tang S, Le SY (2008). Aberrant expression of oncogenic and tumor-suppressive microRNAs in cervical cancer is required for cancer cell growth. *PLoS One*.

[12] Cai X, Li G, Laimins LA, Cullen BR (2006). Human papillomavirus genotype 31 does not express detectable microRNA levels during latent or productive virus replication. *Journal of Virology*.

[13] He L, Thomson JM, Hemann MT (2005). A microRNA polycistron as a potential human oncogene. *Nature*.

[14] He L, He X, Lim LP (2007). A microRNA component of the p53 tumour suppressor network. *Nature*.

[15] Croce CM (2008). Oncogenes and cancer. *The New England Journal of Medicine*.

[16] Córdova-Alarcón E, Centeno F, Reyes-Esparza J, García-Carrancá A, Garrido E (2005). Effects of HRAS oncogene on cell cycle progression in a cervical cancer-derived cell line. *Archives of Medical Research*.

[17] Li Y, Wang F, Xu J (2011). Progressive miRNA expression profiles in cervical carcinogenesis and identification of HPV-related target genes for miR-29. *Journal of Pathology*.

[18] Lui W-O, Pourmand N, Patterson BK, Fire A (2007). Patterns of known and novel small RNAs in human cervical cancer. *Cancer Research*.

[19] Johnson SM, Grosshans H, Shingara J (2005). RAS is regulated by the let-7 microRNA family. *Cell*.

[20] Dufourcq P, Leroux L, Ezan J (2008). Regulation of endothelial cell cytoskeletal reorganization by a secreted frizzled-related protein-1 and frizzled 4- and frizzled 7-dependent pathway: role in neovessel formation. *American Journal of Pathology*.

[21] Minet E, Michel G, Mottet D (2001). C-JUN gene induction and AP-1 activity is regulated by a JNK-dependent pathway in hypoxic HepG2 cells. *Experimental Cell Research*.

[22] Manavi M, Hudelist G, Retter AF, Gschwandtler DK, Pischinger K, Czerwenka K (2007). Gene profiling in Pap-cell smears of high-risk human papillomavirus-positive squamous cervical carcinoma. *Gynecologic Oncology*.

[23] Kottakis F, Polytarchou C, Foltopoulou P, Sanidas I, Kampranis SC, Tsichlis PN (2011). FGF-2 regulates cell proliferation, migration, and angiogenesis through an NDY1/KDM2B-miR-101-EZH2 pathway. *Molecular Cell*.

[24] Varambally S, Cao Q, Mani R-S (2008). Genomic loss of microRNA-101 leads to overexpression of histone methyltransferase EZH2 in cancer. *Science*.

[25] Fang J, Zhang M, Li Q (2011). Enhancer of zeste homolog 2 expression is associated with tumor cell proliferation and invasion in cervical cancer. *American Journal of the Medical Sciences*.

[26] Moore H, Gonzalez M, Toy K, Cimino AM, Argani P, Kleer C (2013). EZH2 inhibition decreases p38 signaling and suppresses breast cancer motility and metastasis. *Breast Cancer Research and Treatment*.

[27] Vire E, Brenner C, Deplus R (2006). The Polycomb group protein EZH2 directly controls DNA methylation. *Nature*.

[28] Holland D, Hoppe KS, Schuller B (2008). Activation of the enhancer of zeste homologue 2 gene by the human papillomavirus E7 oncoprotein. *Cancer Research*.

[29] Negrini M, Calin GA (2008). Breast cancer metastasis: a microRNA story. *Breast Cancer Research*.

[30] Martinez I, Gardiner AS, Board KF, Monzon FA, Edwards RP, Khan SA (2008). Human papillomavirus type 16 reduces the expression of microRNA-218 in cervical carcinoma cells. *Oncogene*.

[31] Liu B, Peng XC, Zheng XL, Wang J, Qin YW (2009). MiR-126 restoration down-regulate VEGF and inhibit the growth of lung cancer cell lines in vitro and in vivo. *Lung Cancer*.

[32] Gaffney DK, Haslam D, Tsodikov A (2003). Epidermal growth factor receptor (EGFR) and vascular endothelial growth factor (VEGF) negatively affect overall survival in carcinoma of the cervix treated with radiotherapy. *International Journal of Radiation Oncology Biology Physics*.

[33] Wang X, Tournier C (2006). Regulation of cellular functions by the ERK5 signalling pathway. *Cellular Signalling*.

[34] Li Y, Zhang M, Chen H (2010). Ratio of miR-196s to HOXC8 messenger RNA correlates with breast cancer cell migration and metastasis. *Cancer Research*.

[35] How C, Hui A, Boutros P (2012). microRNA-196b regulates HOXB7 in cervical cancer. *Cancer Research*.

[36] How C, Hui AB, Fyles AW, Liu FF (2010). The role of microRNA-196b in cervical cancer. *Cancer Research*.

[37] Uesugi A, Kozaki K-I, Tsuruta T (2011). The tumor suppressive microRNA miR-218 targets the mTOR component rictor and inhibits AKT phosphorylation in oral cancer. *Cancer Research*.

[38] Wu DW, Cheng YW, Wang J, Chen CY, Lee H (2010). Paxillin predicts survival and relapse in non-small cell lung cancer by microRNA-218 targeting. *Cancer Research*.

[39] Tatarano S, Chiyomaru T, Kawakami K (2011). miR-218 on the genomic loss region of chromosome 4p15.31 functions as a tumor suppressor in bladder cancer. *International Journal of Oncology*.

[40] Tie J, Pan Y, Zhao L (2010). MiR-218 inhibits invasion and metastasis of gastric cancer by targeting the robo1 receptor. *PLoS Genetics*.

[41] Legg JA, Herbert JMJ, Clissold P, Bicknell R (2008). Slits and Roundabouts in cancer, tumour angiogenesis and endothelial cell migration. *Angiogenesis*.

[42] Wang B, Xiao Y, Ding BB (2003). Induction of tumor angiogenesis by Slit-Robo signaling and inhibition of cancer growth by blocking Robo activity. *Cancer Cell*.

[43] Gardiner AS, McBee WC, Jr. (2011). MicroRNA analysis in human papillomavirus (HPV)-associated cervical neoplasia and cancer. *Journal Carcinogene Mutagene*.

[44] Yu J, Wang Y, Dong R, Huang X, Ding S, Qiu H (2012). Circulating microRNA-218 was reduced in cervical cancer and correlated with tumor invasion. *Journal of Cancer Research and Clinical Oncology*.

[45] Forrest ARR, Kanamori MK, Tomaru Y (2010). Induction of microRNAs, mir-155, mir-222, mir-424 and mir-503, promotes monocytic differentiation through combinatorial regulation. *Leukemia*.

[46] Pallasch CP, Patz M, Yoon JP (2009). miRNA deregulation by epigenetic silencing disrupts suppression of the oncogene PLAG1 in chronic lymphocytic leukemia. *Blood*.

[47] Xu J, Li Y, Wang F (2012). Suppressed miR-424 expression via upregulation of target gene Chk1 contributes to the progression of cervical cancer. *Oncogene*.

[48] Zachos G, Rainey MD, Gillespie DAF (2003). Chk1-deficient tumour cells are viable but exhibit multiple checkpoint and survival defects. *EMBO Journal*.

[49] Volinia S, Calin GA, Liu C-G (2006). A microRNA expression signature of human solid tumors defines cancer gene targets. *Proceedings of the National Academy of Sciences of the United States of America*.

[50] Pereira PM, Marques JP, Soares AR, Carreto L, Santos MAS (2010). Microrna expression variability in human cervical tissues. *PLoS One*.

[51] Long M-J, Wu F-X, Li P, Liu M, Li X, Tang H (2012). MicroRNA-10a targets CHL1 and promotes cell growth, migration and invasion in human cervical cancer cells. *Cancer Letters*.

[52] Zhang J-G, Wang J-J, Zhao F, Liu Q, Jiang K, Yang GH (2010). MicroRNA-21 (miR-21) represses tumor suppressor PTEN and promotes growth and invasion in non-small cell lung cancer (NSCLC). *Clinica Chimica Acta International Journal of Clinical Chemistry*.

[53] Hui ABY, Lenarduzzi M, Krushel T (2010). Comprehensive MicroRNA profiling for head and neck squamous cell carcinomas. *Clinical Cancer Research*.

[54] Meng F, Henson R, Wehbe HJ, Ghoshal K, Jacob ST, Patel T (2007). MicroRNA-21 regulates expression of the PTEN tumor suppressor gene in human hepatocellular cancer. *Gastroenterology*.

[55] Zhai Y, Hotary KB, Nan B (2005). Expression of membrane type 1 matrix metalloproteinase is associated with cervical carcinoma progression and invasion. *Cancer Research*.

[56] Hagemann T, Bozanovic T, Hooper S (2007). Molecular profiling of cervical cancer progression. *British Journal of Cancer*.

[57] Zhu S, Si ML, Wu H, Mo YY (2007). MicroRNA-21 targets the tumor suppressor gene tropomyosin 1 (TPM1). *Journal of Biological Chemistry*.

[58] Zhu S, Wu H, Wu F, Nie D, Sheng S, Mo YY (2008). MicroRNA-21 targets tumor suppressor genes in invasion and metastasis. *Cell Research*.

[59] Wong Y-F, Cheung T-H, Tsao GSW (2006). Genome-wide gene expression profiling of cervical cancer in Hong Kong women by oligonucleotide microarray. *International Journal of Cancer*.

[60] Yao Q, Xu H, Zhang QQ, Zhou H, Qu LH (2009). MicroRNA-21 promotes cell proliferation and down-regulates the expression of programmed cell death 4 (PDCD4) in HeLa cervical carcinoma cells. *Biochemical and Biophysical Research Communications*.

[61] Pan S, Yu F, Gong C, Song E (2009). Tumor invasion and metastasis initiated by mir-106b in breast cancer by targeting BRMS1 and RB. *Cancer Research*.

[62] Shai A, Brake T, Somoza C, Lambert PF (2007). The human papillomavirus E6 oncogene dysregulates the cell cycle and contributes to cervical carcinogenesis through two independent activities. *Cancer Research*.

[63] Balsitis S, Dick F, Dyson N, Lambert PF (2006). Critical roles for non-pRb targets of human papillomavirus type 16 E7 in cervical carcinogenesis. *Cancer Research*.

[64] Araki T, Milbrandt J (2000). Ninjurin2, a novel homophilic adhesion molecule, is expressed in mature sensory and enteric neurons and promotes neurite outgrowth. *Journal of Neuroscience*.

[65] Gius D, Funk MC, Chuang EY (2007). Profiling microdissected epithelium and stroma to model genomic signatures for cervical carcinogenesis accommodating for covariates. *Cancer Research*.

[66] Wang YX, Zhang XY, Zhang BF, Yang CQ, Chen XM, Gao HJ (2010). Initial study of microRNA expression profiles of colonic cancer without lymph node metastasis. *Journal of Digestive Diseases*.

[67] Descargues P, Draison C, Bonnart C (2005). Spink5-deficient mice mimic Netherton syndrome through degradation of desmoglein 1 by epidermal protease hyperactivity. *Nature Genetics*.

[68] Lee J-W, Park Y-A, Choi J-J (2011). The expression of the miRNA-200 family in endometrial endometrioid carcinoma. *Gynecologic Oncology*.

[69] Porkka KP, Pfeiffer MJ, Waltering KK, Vessella RL, Tammela TLJ, Visakorpi T (2007). MicroRNA expression profiling in prostate cancer. *Cancer Research*.

[70] Iorio MV, Visone R, Di Leva G (2007). MicroRNA signatures in human ovarian cancer. *Cancer Research*.

[71] Wang B, Koh P, Winbanks C (2011). MiR-200a prevents renal fibrogenesis through repression of TGF-*β*2 expression. *Diabetes*.

[72] Xu X-C, Mitchell MF, Silva E, Jetten A, Lotan R (1999). Decreased expression of retinoic acid receptors, transforming growth factor *β*, involucrin, and cornifin in cervical intraepithelial neoplasia. *Clinical Cancer Research*.

[73] Budhu A, Ji J, Wang XW (2010). The clinical potential of microRNAs. *Journal of Hematology and Oncology*.

[74] Guo S-L, Peng Z, Yang X (2011). mir-148a promoted cell proliferation by targeting p27 in gastric cancer cells. *International Journal of Biological Sciences*.

[75] De Putte GV, Holm R, Lie AK, Tropé CG, Kristensen GB (2003). Expression of p27, p21, and p16 protein in early squamous cervical cancer and its relation to prognosis. *Gynecologic Oncology*.

[76] Bernard S, Eilers M (2006). Control of cell proliferation and growth by Myc proteins. *Results and Problems in Cell Differentiation*.

[77] Zhang Z, Sun H, Dai H (2009). MicroRNA miR-210 modulates cellular response to hypoxia through the MYC antagonist MNT. *Cell Cycle*.

[78] Arvanitis DA, Spandidos DA (2008). Deregulation of the G1/S phase transition in cancer and squamous intraepithelial lesions of the uterine cervix: a case control study. *Oncology Reports*.

[79] Yang H, Kong W, He L (2008). MicroRNA expression profiling in human ovarian cancer: miR-214 induces cell survival and cisplatin resistance by targeting PTEN. *Cancer Research*.

[80] Lee J-W, Choi CH, Choi J-J (2008). Altered MicroRNA expression in cervical carcinomas. *Clinical Cancer Research*.

[81] Lee JS, Choi YD, Lee JH (2006). Expression of PTEN in the progression of cervical neoplasia and its relation to tumor behavior and angiogenesis in invasive squamous cell carcinoma. *Journal of Surgical Oncology*.

[82] Ma YY, Wei SJ, Lin YC (2000). PIK3CA as an oncogene in cervical cancer. *Oncogene*.

[83] Chen C-Z, Li L, Lodish HF, Bartel DP (2004). MicroRNAs modulate hematopoietic lineage differentiation. *Science*.

[84] Wan H, Yuan M, Simpson C (2007). Stem/progenitor cell-like properties of desmoglein 3dim cells in primary and immortalized keratinocyte lines. *Stem Cells*.

[85] Bernards A, Settleman J (2009). Loss of the ras regulator RASAL1: another route to ras activation in colorectal cancer. *Gastroenterology*.

[86] Zheng ZM, Baker CC (2006). Papillomavirus genome structure, expression, and post-transcriptional regulation. *Frontiers in Bioscience*.

[87] Berrington De González A, Green J (2007). Comparison of risk factors for invasive squamous cell carcinoma and adenocarcinoma of the cervix: Collaborative reanalysis of individual data on 8, 097 women with squamous cell carcinoma and 1, 374 women with adenocarcinoma from 12 epidemiological studies. *International Journal of Cancer*.

[88] Castellsagué X, Diaz M, de Sanjosé S (2006). Worldwide human papillomavirus etiology of cervical adenocarcinoma and its cofactors: implications for screening and prevention. *Journal of the National Cancer Institute*.

[89] Wang X, Wang HK, Mccoy JP (2009). Oncogenic HPV infection interrupts the expression of tumor-suppressive miR-34a through viral oncoprotein E6. *RNA*.

[90] Thorland EC, Myers SL, Gostout BS, Smith DI (2003). Common fragile sites are preferential targets for HPV16 integrations in cervical tumors. *Oncogene*.

[91] Yao T, Lin Z (2012). MiR-21 is involved in cervical squamous cell tumorigenesis and regulates CCL20. *Biochimica et Biophysica Acta*.

[92] Nominé Y, Masson M, Charbonnier S (2006). Structural and functional analysis of E6 oncoprotein: insights in the molecular pathways of human papillomavirus-mediated pathogenesis. *Molecular Cell*.

[93] Au Yeung CL, Tsang TY, Yau PL, Kwok TT (2011). Human papillomavirus type 16 E6 induces cervical cancer cell migration through the p53/microRNA-23b/urokinase-type plasminogen activator pathway. *Oncogene*.

[94] Duffy MJ, Maguire TM, McDermott EW, 'Higgins NO (1999). Urokinase plasminogen activator: a prognostic marker in multiple types of cancer. *Journal of Surgical Oncology*.

[95] Riethdorf L, Riethdorf S, Petersen S (1999). Urokinase gene expression indicates early invasive growth in squamous cell lesions of the uterine cervix. *The Journal of Pathology*.

[96] Chang T-C, Wentzel EA, Kent OA (2007). Transactivation of miR-34a by p53 broadly influences gene expression and promotes apoptosis. *Molecular Cell*.

[97] Raver-Shapira N, Marciano E, Meiri E (2007). Transcriptional activation of miR-34a contributes to p53-mediated apoptosis. *Molecular Cell*.

[98] Sun F, Fu H, Liu Q (2008). Downregulation of CCND1 and CDK6 by miR-34a induces cell cycle arrest. *FEBS Letters*.

[99] Wang X, Meyers C, Guo M, Zheng ZM (2011). Upregulation of p18Ink4c expression by oncogenic HPV E6 via p53-miR-34a pathway. *International Journal of Cancer*.

[100] Myklebust MP, Bruland O, Fluge O, Skarstein A, Balteskard L, Dahl O (2011). MicroRNA-15b is induced with E2F-controlled genes in HPV-related cancer. *British Journal of Cancer*.

[101] Gonzalez SL, Stremlau M, He X, Basile JR, Münger K (2001). Degradation of the retinoblastoma tumor suppressor by the human papillomavirus type 16 E7 oncoprotein is important for functional inactivation and is separable from proteasomal degradation of E7. *Journal of Virology*.

[102] Dyson N, Howley PM, Munger K, Harlow E (1989). The human papilloma virus-16 E7 oncoprotein is able to bind to the retinoblastoma gene product. *Science*.

[103] Ofir M, Hacohen D, Ginsberg D (2011). miR-15 and miR-16 are direct transcriptional targets of E2F1 that limit E2F-induced proliferation by targeting cyclin E. *Molecular Cancer Research*.

[104] Bueno MJ, De Cedrón MG, Laresgoiti U, Fernández-Piqueras J, Zubiaga AM, Malumbres M (2010). Multiple E2F-induced microRNAs prevent replicative stress in response to mitogenic signaling. *Molecular and Cellular Biology*.

[105] da Silva Cardeal LB, Boccardo E, Termini L (2012). HPV16 oncoproteins induce MMPs/RECK-TIMP-2 imbalance in primary keratinocytes: possible implications in cervical carcinogenesis. *PLoS One*.

[106] Takagi S, Simizu S, Osada H (2009). Reck negatively regulates matrix metalloproteinase-9 transcription. *Cancer Research*.

[107] Sasahara RM, Brochado SM, Takahashi C (2002). Transcriptional control of the RECK metastasis/angiogenesis suppressor gene. *Cancer Detection and Prevention*.

[108] Yi R, Poy MN, Stoffel M, Fuchs E (2008). A skin microRNA promotes differentiation by repressing ‘stemness’. *Nature*.

[109] Melar-New M, Laimins LA (2010). Human papillomaviruses modulate expression of microRNA 203 upon epithelial differentiation to control levels of p63 proteins. *Journal of Virology*.

[110] Lena AM, Shalom-Feuerstein R, di Val Cervo PR (2008). miR-203 represses “stemness” by repressing ΔNp63. *Cell Death and Differentiation*.

[111] Truong AB, Kretz M, Ridky TW, Kimmel R, Khavari PA (2006). p63 regulates proliferation and differentiation of developmentally mature keratinocytes. *Genes and Development*.

[112] Spitkovsky D, Hehner SP, Hofmann TG, Möller A, Lienhard Schmitz M (2002). The human papillomavirus oncoprotein E7 attenuates NF-*κ*B activation by targeting the I*κ*B kinase complex. *Journal of Biological Chemistry*.

[113] McKenna DJ, McDade SS, Patel D, McCance DJ (2010). MicroRNA 203 expression in keratinocytes is dependent on regulation of p53 levels by E6. *Journal of Virology*.

[114] Ferretti E, De Smaele E, Po A (2009). MicroRNA profiling in human medulloblastoma. *International Journal of Cancer*.

[115] Gaur A, Jewell DA, Liang Y (2007). Characterization of microRNA expression levels and their biological correlates in human cancer cell lines. *Cancer Research*.

[116] Katada T, Ishiguro H, Kuwabara Y (2009). MicroRNA expression profile in undifferentiated gastric cancer. *International Journal of Oncology*.

[117] Bandrés E, Cubedo E, Agirre X (2006). Identification by Real-time PCR of 13 mature microRNAs differentially expressed in colorectal cancer and non-tumoral tissues. *Molecular Cancer*.

[118] Kong W, Yang H, He L (2008). MicroRNA-155 is regulated by the transforming growth factor *β*/Smad pathway and contributes to epithelial cell plasticity by targeting RhoA. *Molecular and Cellular Biology*.

[119] Perez-Moreno M, Jamora C, Fuchs E (2003). Sticky business: orchestrating cellular signals at adherens junctions. *Cell*.

[120] Peralta-Zaragoza O, Bermúdez-Morales V, Gutiérrez-Xicotencatl L, Alcocer-González J, Recillas-Targa F, Madrid-Marina V (2006). E6 and E7 oncoproteins from human papillomavirus type 16 induce activation of human transforming growth factor *β*1 promoter throughout Sp1 recognition sequence. *Viral Immunology*.

[121] Noordhuis MG, Fehrmann RSN, Wisman GBA (2011). Involvement of the TGF-*β* and *β*-catenin pathways in pelvic lymph node metastasis in early-stage cervical cancer. *Clinical Cancer Research*.

[122] Ahn WS, Bae SM, Lee JM (2004). Searching for pathogenic gene functions to cervical cancer. *Gynecologic Oncology*.

[123] Iorio MV, Ferracin M, Liu CG (2005). MicroRNA gene expression deregulation in human breast cancer. *Cancer Research*.

[124] Maufort JP, Shai A, Pitot HC, Lambert PF (2010). A role for HPV16 E5 in cervical carcinogenesis. *Cancer Research*.

[125] Kivi N, Greco D, Auvinen P, Auvinen E (2008). Genes involved in cell adhesion, cell motility and mitogenic signaling are altered due to HPV 16 E5 protein expression. *Oncogene*.

[126] Riley RR, Duensing S, Brake T, Münger K, Lambert PF, Arbeit JM (2003). Dissection of human papillomavirus E6 and E7 function in transgenic mouse models of cervical carcinogenesis. *Cancer Research*.

[127] Greco D, Kivi N, Qian K, Leivonen S-K, Auvinen P, Auvinen E (2011). Human papillomavirus 16 E5 modulates the expression of host microRNAS. *PLoS One*.

[128] Sabatini PJB, Zhang M, Silverman-Gavrila R, Bendeck MP, Langille BL (2008). Homotypic and endothelial cell adhesions via N-cadherin determine polarity and regulate migration of vascular smooth muscle cells. *Circulation Research*.

[129] Mani SA, Guo W, Liao M-J (2008). The epithelial-mesenchymal transition generates cells with properties of stem cells. *Cell*.

[130] Pim D, Bergant M, Boon SS (2012). Human papillomaviruses and the specificity of PDZ domain targeting. *The FEBS Journal*.

[131] Gantier MP, McCoy CE, Rusinova I (2011). Analysis of microRNA turnover in mammalian cells following Dicer1 ablation. *Nucleic Acids Research*.

[132] Chen X, Ba Y, Ma L (2008). Characterization of microRNAs in serum: a novel class of biomarkers for diagnosis of cancer and other diseases. *Cell Research*.

[133] Li J, Smyth P, Flavin R (2007). Comparison of miRNA expression patterns using total RNA extracted from matched samples of formalin-fixed paraffin-embedded (FFPE) cells and snap frozen cells. *BMC Biotechnology*.

[134] Lee J-W, Choi CH, Choi JJ (2008). Altered microRNA expression in cervical carcinomas. *Clinical Cancer Research*.

[135] Calin GA, Ferracin M, Cimmino A (2005). A microRNA signature associated with prognosis and progression in chronic lymphocytic leukemia. *The New England Journal of Medicine*.

[136] Roldo C, Missiaglia E, Hagan JP (2006). MicroRNA expression abnormalities in pancreatic endocrine and acinar tumors are associated with distinctive pathologic features and clinical behavior. *Journal of Clinical Oncology*.

[137] Blenkiron C, Goldstein LD, Thorne NP (2007). MicroRNA expression profiling of human breast cancer identifies new markers of tumor subtype. *Genome Biology*.

[138] Raponi M, Dossey L, Jatkoe T (2009). MicroRNA classifiers for predicting prognosis of squamous cell lung cancer. *Cancer Research*.

[139] Hu X, Schwarz JK, Lewis JS (2010). A microRNA expression signature for cervical cancer prognosis. *Cancer Research*.

[140] Heiden MGV, Cantley LC, Thompson CB (2009). Understanding the warburg effect: the metabolic requirements of cell proliferation. *Science*.

[141] Gee HE, Camps C, Buffa FM (2010). hsa-mir-210 is a marker of tumor hypoxia and a prognostic factor in head and neck cancer. *Cancer*.

